# Developing Antiviral Vaccines Based on Attenuated *Salmonella*

**DOI:** 10.3390/pathogens15090955

**Published:** 2026-09-08

**Authors:** Ethan Ou, Fenyong Liu

**Affiliations:** 1 School of Public Health, University of California, Berkeley, CA 94720, USA; 2 Program in Comparative Biochemistry, University of California, Berkeley, CA 94720, USA

**Keywords:** antiviral, gene delivery, gene therapy, *Salmonella*, vaccine

## Abstract

Attenuated *Salmonella* strains are a promising gene delivery vector in the development of vaccines targeting various human diseases. For example, the Ty21a vaccine is a *Salmonella*-based vaccine that elicits a robust local, cellular, and systemic immune response against *Salmonella typhi* infection. Human immunodeficiency virus (HIV) is responsible for the AIDS pandemic and requires lifelong treatment to manage. Human papillomavirus (HPV) is the cause of the vast majority of cervical cancer cases in the global population. Influenza is a seasonal, pandemic virus that can lead to high death tolls in humans and livestock and considerable financial losses. In this review, we outline current methods of protection and treatment for HIV, HPV, and influenza, as well as how a versatile *Salmonella*-based gene delivery vector’s mechanism of action can lead to immunity. We also describe several studies that utilize many of these *Salmonella*-based vector vaccines to provide protection against infection with these viruses, as many of these *Salmonella*-based vectors exhibit different factors that may enhance their gene delivery, immune responses, and post-delivery clearance out of host cells. Finally, we discuss the benefits and drawbacks of using *Salmonella*-based vaccines and future directions for these vaccine candidates, as well as the implications of introducing these vaccines into the current landscape.

## 1. Introduction

*Salmonella* is a Gram-negative facultative anaerobe that is typically a foodborne pathogen in humans [1]. The primary mechanism of infection of this bacterium is through intracellular invasion of cells in the human gut [2]. Upon infection, *Salmonella* generally causes gastroenteritis and typhoid fever [3]. Although *Salmonella* is usually seen as something that should be avoided, recent advances in vaccine development for various diseases have transformed this bacterium into a powerful vector for delivery of genetic material encoding antigens to elicit strong immune responses [4]. These live attenuated *Salmonella*-based vaccine vectors have shown great potential in their ability to deliver therapeutic elements intracellularly to provide adequate protection against infection of numerous pathogens, including human immunodeficiency virus, human papillomavirus, and influenza.

Human immunodeficiency virus (HIV) is responsible for all cases of acquired immunodeficiency syndrome (AIDS) [5]. It is part of the *Retroviridae* family, a large group of enveloped viruses containing single-stranded RNA genomes that use reverse transcriptase and integrase to reverse-transcribe their genome into double-stranded DNA and embed it into the host genome, respectively [6]. HIV belongs to a genus of retroviruses known as lentiviruses, which are unique for their long incubation periods and ability to infect non-dividing cells, leading to lifelong infection [7]. In 2024, 40.8 million people were estimated to be living with HIV, with an estimated 630,000 deaths related to HIV infection [8]. The AIDS pandemic was officially recognized in 1981; however, it likely began to spread in western Africa from the years 1910 to 1930 [9]. HIV infection causes significant immune system suppression in hosts, particularly by targeting CD4+ T cells and causing profound loss of these immune cells [10]. Once HIV infection progresses to AIDS, which is the symptomatic manifestation of the infection, individuals become susceptible to opportunistic infections from various other pathogens. Currently, cART (combination antiretroviral therapy) remains the most widespread treatment of HIV infection [11].

Human papillomavirus (HPV) is the most common sexually transmitted disease in humans and is the leading cause of cervical cancer, lending it the title of an oncogenic virus [12]. HPV belongs to the *Papillomaviridae* family, which is a large family of non-enveloped, circular, double-stranded DNA viruses [13]. There are over 200 types of HPV with varying levels of burden of disease. Many HPV infections are classified as low-risk and, in healthy adults, can be resolved by the immune system. Low-risk genotypes of HPV include HPV 6, 11, 42, 43, and 44. However, infection with high-risk HPVs may cause oral or genital lesions, and persistent high-risk infection may cause individuals to develop cervical cancer or other anogenital cancers [14]. High-risk genotypes include HPV 16, 18, 31, 33, 34, 35, 39, 45, 51, 52, 56, 58, 59, 66, 68, and 70 [15]. Globally, there were an estimated 620,000 cases of cancer caused by HPV infection in women and 70,000 cases in men in 2019 [16].

Influenza is one of the most prevalent seasonal infectious diseases in the world. Responsible for numerous pandemics throughout human history, it continues to engage vaccine developers in a decades-long arms race. This is due to the rapidly evolving nature of influenza, which is the result of the structure of its genome. Influenza belongs to the *Orthomyxoviridae* family of viruses, which consists of enveloped viruses with segmented, negative-sense, single-stranded RNA genomes [17]. The segmented nature of the genome allows for a process called reassortment to occur, which is the shuffling of genes between different strains of influenza upon co-infection.

In this review, we will be discussing the recent developments in *Salmonella*-based vaccines against human immunodeficiency virus, human papillomavirus, and influenza. We will outline the routes of administration, mechanisms of delivery, and the strength of immune responses elicited by these vaccines, advantages of such vaccines, and future directions.

## 2. Current Vaccines and Therapeutics

### 2.1. HIV

Currently, there are no FDA-approved vaccines against HIV. However, there have been clinical trials with varying results and conclusions. Many of these clinical trials were halted due to the lack of efficacy in the vaccines [18].

Despite the difficulties in developing an effective vaccine, some trials have shown promising results that could give strong insight into what HIV vaccines must accomplish to provide strong protection. A 2021 study showed that a vaccine that promotes the production of broadly neutralizing antibodies (bnAbs) may be a promising route [19]. The trial included 48 HIV-negative adults who were immunized with the eOD-GT8 60mer vaccine, resulting in 97% of participants developing precursors to broadly neutralizing VRC01-class antibodies. These antibodies are one of several antibodies generated against the gp120 envelope glycoprotein, which is crucial for entry of HIV into host cells through binding to CD4 [20]. Boost immunization may further increase this vaccine’s efficacy, as seen in a 2024 study involving core-g28v2 60mer, which is a nanoparticle immunogen, and has already shown increased VRC01 precursor diversity, affinity, and frequency in humanized mice [21].

A 2025 trial showed reassuring results, using a vaccine containing a glucopyranosyl lipid A-stable emulsion (GLA-SE) adjuvant with a CH505 transmitter founder (CH505TF) gp120 immunogen [22]. The trial enrolled 38 infants who were born to mothers living with HIV and were administered either the vaccine or a placebo. Infants were chosen as the testing population due to their increased ability to generate broadly neutralizing antibodies (bnAbs), which is a major goal for many potential HIV vaccines. The trial showed that those given the vaccine had higher rates of local and systemic reactions compared to those given the placebo, with both groups showing little to no adverse effects. Although the data in this trial did not show significant differences in the reactions to the vaccine, it illustrated that adjuvanted HIV vaccines in exposed infants may be a feasible route of protection.

The leading treatment for those affected by HIV is antiretroviral therapy (ART). This line of treatment involves a combination of many different drugs that manage and suppress the replication of HIV in a patient’s body [23]. While it is unlikely that ART can cure someone with HIV infection, with strict drug adherence, it has the potential to provide lifelong relief. The classes of drugs included in ART are reverse transcriptase inhibitors, protease inhibitors, fusion inhibitors, chemokine receptor 5 antagonists, and integrase strand transfer inhibitors [24]. ART has greatly increased the life expectancy of HIV-infected individuals, potentially adding decades to patients’ lives with consistent usage of ART for the rest of their lives [25].

### 2.2. HPV

There are currently three FDA-approved HPV vaccines that provide strong protection against HPV, each requiring multiple doses for maximum efficacy. The three vaccines are the quadrivalent vaccine, the bivalent vaccine, and the nine-valent vaccine [26].

The quadrivalent vaccine, Gardasil, was the first commercially available HPV vaccine, being licensed in 2006 by the FDA. It is a recombinant vaccine that contains a mixture of virus-like particles from L1 capsid proteins that confers protection against HPV types 6, 11, 16, and 18 [27]. Currently, the vaccine is administered intramuscularly on a three-dose schedule at 0, 2, and 6 months old. It has shown great efficacy, being more than 90% effective in preventing cervical intraepithelial neoplasia, adenocarcinoma in situ, anogenital and vaginal lesions, and cervical lesions, showing great protection against HPV infection [28].

Similar to the quadrivalent vaccine, the bivalent HPV vaccine (Cervavix) also contains virus-like particles but focuses on the HPV types 16 and 18, which are responsible for about 70% of cervical cancer cases globally [29]. It was approved by the FDA in 2009 and is administered at 0, 1, and 6 months on a three-dose schedule [26]. The bivalent vaccine showed a comparable 94.9% cumulative efficacy to the quadrivalent vaccine, showing high efficacy against HPV16/18-associated precancer for over a decade after initial vaccination [30].

Gardasil 9, approved by the FDA in 2014, follows the same administration schedule as the quadrivalent vaccine and contains virus-like particles for HPV 6, 11, 16, 18, 31, 33, 45, 52, and 58 [26]. In addition to similar protection against HPV 6, 11, 16, and 18 to the quadrivalent vaccine, this vaccine prevented infection, lesions, and other cytological abnormalities associated with the five other genotypes not covered by the quadrivalent vaccine. This all-encompassing approach resulted in a 97.4 overall efficacy in this vaccine, due to its wide coverage of HPV genotypes [31].

### 2.3. Influenza

There are currently three types of vaccines against influenza licensed in various countries: inactivated, live attenuated, and recombinant HA vaccines [32]. Due to the rapid evolution of influenza, doses of vaccines must be administered annually to maintain maximum protection against present strains.

Inactivated vaccines (IVs), which can be further classified into whole, split, and subunit IVs, are generally produced by growing the virus in embryonated chicken eggs and then inactivating them in formaldehyde or β-propiolactone [33]. These vaccines are typically administered via intramuscular injection. Although the virus is inactivated, it still has the ability to induce an immune response because it still carries the necessary epitopes to do so [34].

The only FDA-approved live attenuated influenza vaccine (LAIV) in the United States (FluMist) is administered intranasally. These vaccines are developed through genetic modifications that decrease virulence while still potentially allowing the virus to persist [35]. Live attenuated vaccines mimic the mechanisms of a natural infection, which could lead to a more robust and longer immune response [32].

Recombinant vaccines contain purified viral antigens, often produced by recombinant DNA, and are usually administered intramuscularly [35]. These vaccines are produced using baculovirus expression vector systems (BEVs), where insect cells are infected with a baculovirus vector containing recombinant DNA expressing influenza viral proteins [36].

## 3. Introduction to Salmonella-Based Vectors

### 3.1. Salmonella Pathogenicity

**Figure 1:**
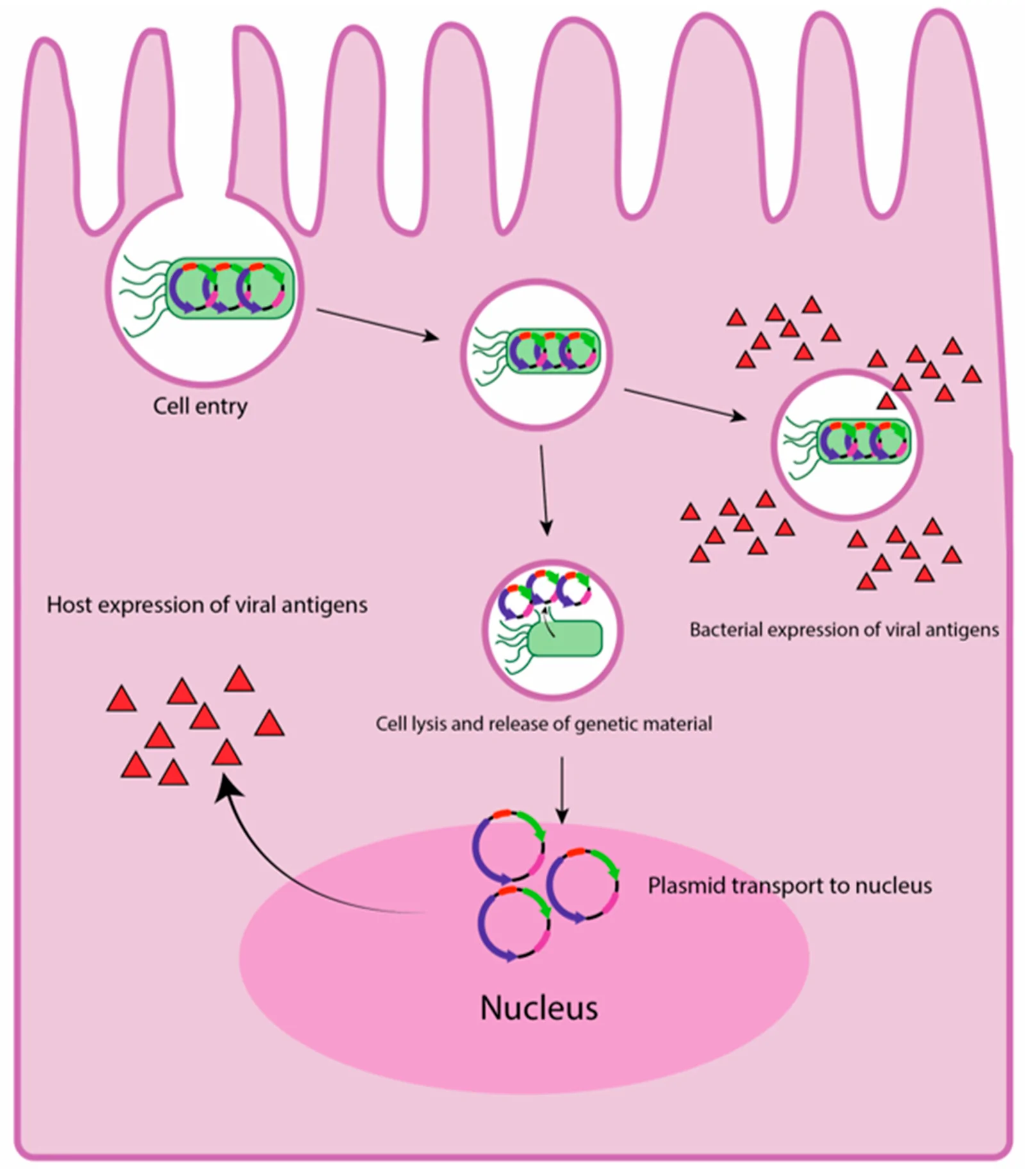
Schematic representation of *Salmonella*-based vector vaccine antigen (denoted by red triangles) expression through host and bacterial means. Attenuated *Salmonella* enters host enterocytes and either directly expresses viral antigens or undergoes lysis, which releases plasmid DNA to be trafficked into the nucleus for transcription and eventual expression of viral antigens. Viral antigens elicit mucosal and systemic immune responses.

*Salmonella* is an intracellular pathogen, and infection primarily occurs through ingestion of contaminated food. *Salmonella* generally invades intestinal epithelial cells (Figure 1), especially cells that reside in the Peyer’s patches of the small intestine [37]. Upon entry into a host cell, *Salmonella* relies on its ability to establish *Salmonella*-containing vacuoles (SCVs), which are compartments surrounded by membranes where they predominantly reside and multiply [38].

*Salmonella* pathogenicity also depends on the expression of two Type 3 Secretion Systems (T3SS), aptly named T3SS1 and T3SS2, which translocate bacterial effector proteins [39]. Interestingly, the functions of these T3SSs can be leveraged to effectively translocate fusion proteins of effector proteins with antigens into the cytoplasm of a host cell. This efficient translocation method can lead to a host cell immune response through the MHC class I pathway [40].

T3SS1 is encoded by *Salmonella* pathogenicity island 1 (SPI-1), which is responsible for transcription factors that regulate various virulence factors [41]. T3SS2 is encoded by *Salmonella* pathogenicity island 2 (SPI-2), which is generally associated with functions related to intracellular growth and proliferation, as opposed to SPI-1 primarily being involved in the invasion of epithelial cells; however, their functions often overlap and complement each other to aid in overall *Salmonella* survival [42]. The T3SS1 effectors SipA, SopA, SopB, and SpiC/SsaB are generally responsible for the early development of SCV formation. SipA specifically works with T3SS2 effector SifA to maintain SCV positioning. Other T3SS2 effectors such as PipB2, SopD2, SseF, SseG, SseJ/SifC, and SteC play important roles in SCV integrity, positioning, and motility [43].

### 3.2. Attenuation of Salmonella

**Figure 2:**
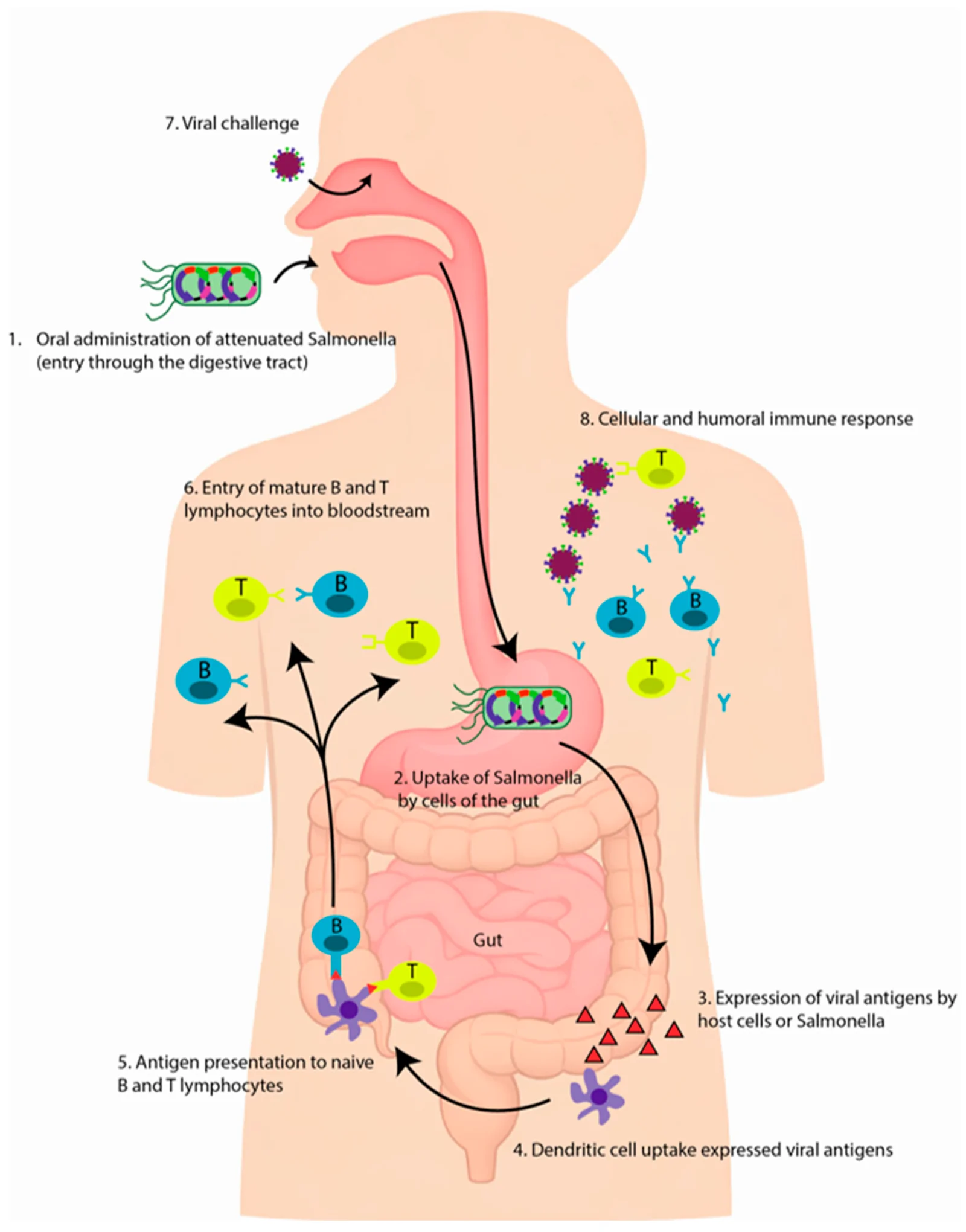
Schematic representation of mucosal and systemic immune responses elicited by oral intake of attenuated *Salmonella* (1). Following *Salmonella* entry into gut epithelial cells (2), expression of viral antigens (denoted by red triangles) (3) allows cells in GALT to phagocytose (4) and present antigens to naïve B and T lymphocytes (5), which will enter the bloodstream as mature lymphocytes (6). Upon viral challenge (7), the immune response will take place at the site of infection (8) and provide protection against the virus.

*Salmonella* vectors in vaccines work as gene delivery methods, usually in the form of DNA plasmids. These plasmids are transformed into the attenuated vectors and cause the expression of antigens of a particular pathogen. Once these attenuated strains of *Salmonella* invade host cells, the antigens expressed by these strains may be detected by host immune cells (Figure 2). Alternatively, some strains are engineered to lyse upon entry into host cells, releasing the genetic material packaged inside, to then be transported into the cell’s nucleus, where it would be transcribed and expressed by host mechanisms (Figure 1). Plasmid DNA released intracellularly from bacterial vectors faces several barriers to expression, such as degradation by nucleases, movement through the cytoplasm, and passage through the nuclear envelope. These challenges may be circumvented through the inclusion of multiple nuclear-targeting sequences for efficient nuclear trafficking and protection from host degradation [44].

Attenuation of *Salmonella* strains generally consists of key deletions of genes that are essential for their survival and pathogenicity. Many attenuated strains of *Salmonella* that were widely used in vaccines incorporated deletions in genes involved in the production of essential aromatic amino acids, such as *aroA*, *aroC*, and *aroD* [45]. These mutations make strains auxotrophic, meaning they cannot synthesize compounds that are integral for their survival, resulting in decreased virulence and making them prime vectors for therapeutic delivery. PhoP/PhoQ mutations are also strong methods of attenuation, as they disable the PhoP/PhoQ regulon, which modulates environmental tolerance genes, virulence factors, and other genes related to invasion and anti-microbial resistance [46]. There are several other approaches of genetically engineered mutagenesis, all with the goal of decreasing the intracellular virulence of *Salmonella* [47].

### 3.3. Comparison with Other Genetic Delivery Platforms

The intracellular delivery pathways of mRNA and adenoviral vector vaccines differ substantially from the *Salmonella*-based DNA delivery methods described above. mRNA vaccines typically contain lipid nanoparticle-encapsulated mRNA that releases into the cytoplasm, where it can be directly translated without requiring trafficking to the nucleus, avoiding a major intracellular barrier encountered by DNA-based vaccine platforms [48]. Much like *Salmonella* vectors, adenoviral vectors deliver DNA that must reach the host cell’s nucleus for transcription. Adenoviral capsids traffic to the nuclear pore complex, where viral DNA is released for nuclear import and expression [49].

The immune responses induced by oral *Salmonella*-based vaccines also differ from those commonly elicited by mRNA and adenoviral vector vaccines. Adenoviral vector and mRNA vaccines can generate strong systemic humoral and cell-mediated responses [50,51]. In comparison, oral *Salmonella* vectors can generate mucosal immunity, which may be particularly advantageous for viruses that initially infect mucosal surfaces, as well as the ability to elicit robust systemic responses [52].

### 3.4. Typhoid Vaccine Live Oral Ty21a

Ty21a is the only FDA-approved vaccine that utilizes a live attenuated *Salmonella* vector as its main gene delivery method. It is an oral vaccine that protects against typhoid fever and was first developed in the early 1970s [53]. The strain of *Salmonella* used in this vaccine was modified using chemical mutagenesis, causing deficiencies in UDP-galactose-4-epimerase activity [54]. Ty21a requires four doses for maximum efficacy and requires booster vaccinations every 5–7 years to maintain adequate (60–80% efficacy) protection, while producing strong mucosal immune responses, particularly with multifunctional effectors in CD8+ T cells [55]. Mechanistically, Ty21a promotes the expression of the mucosal homing receptor α4β7, allowing local immune responses to mount at the site of infection, typically in the digestive tract upon ingestion [56]. This vaccine can be administered to children as young as 2–3 years old and is a recommended immunization for people who are traveling to regions with higher risk of typhoid fever [57]. The vaccine is not recommended for patients with hypersensitivities to any component of the vaccine, those experiencing acute febrile and gastrointestinal illness, and people who are immunocompromised [58].

An early study with 155 human male volunteers established the safety and efficacy of the Ty21a strain [59]. These volunteers received 5–8 doses of the vaccine without significant side effects, exhibiting low levels of the Ty21a strain excreted in stool generally lasting only 1 day after vaccination. Out of the 958 stool isolates tested, none of them showed reversion to galactose fermentation, and vaccinated volunteers were also significantly protected during subsequent challenge with virulent *S. typhi* [59].

Another study included a total of 32,388 children divided into a placebo control group and a group receiving three doses of the Ty21a vaccine. Results of the study showed a 95% reduction in typhoid incidence during three years of surveillance [60]. Following encouraging early volunteer and field studies, a large randomized controlled trial involving over 100,000 school-aged children in Santiago, Chile, demonstrated sustained protection following oral administration of enteric-coated Ty21a capsules. The results of the study showed a 67% efficacy for at least three years without adverse reactions [61].

One of the first studies to test Ty21a as a gene delivery vector in human volunteers utilized an engineered Ty21a vector to express the UreA and UreB subunits of *Helicobacter pylori*, producing the recombinant strain Ty21a (pDB1) [62]. Nine volunteers received the Ty21a (pDB1) vaccine while three received a placebo, and only three of the nine volunteers produced a weak but significant T-cell response against *H. pylori* urease [62]. A similar study involving 58 human volunteers tested a Ty21a strain expressing *H. pylori* urease, with 13 volunteers showing negative urea breath tests. These included five who had completely cleared *H. pylori* and eight with substantially reduced bacterial burden [63]. While these studies showed Ty21a’s potential to deliver a foreign antigen to humans and its established safety, simply expressing a heterologous antigen in Ty21a did not automatically produce a strong antigen-specific immune response.

While research has shown that prior exposure to Ty21a did not significantly enhance the response to the heterologous urease antigen [64], a four-dose regimen also showed optimal protection and duration of immunity against typhoid fever [65].

### 3.5. Experimental Salmonella-Based Vaccines Against Other Human Diseases

Although there is only one licensed *Salmonella*-based vaccine, there are many other studies that use attenuated *Salmonella* vectors to provide protection against various human diseases through strong mucosal and systemic immune responses (Figure 2). *Salmonella* vectors have been a prime candidate to deliver antigens of many different strains of bacteria such as *Escherichia coli*, *Helicobacter pylori*, *Shigella dysenteriae*, *Yersinia pestis*, and *Mycobacterium tuberculosis* [66]. *Salmonella*-based vaccines have been developed to treat different forms of cancer, as well as the attenuated *Salmonella* strain MvP728, which has been used to deliver tumor-associated antigens and induce anti-tumor activity in mice [67,68]. Similarly, an attenuated *Salmonella* strain, CVD 915, was found to decrease the number of mice liver metastases while inducing a strong cellular immune response [69]. There have been many recent studies that utilized the attenuated *Salmonella* model to generate vaccines against murine cytomegalovirus (MCMV), to show potential avenues for protection against human cytomegalovirus (HCMV). The vectors generated in these studies expressed various MCMV proteins associated with virulence and pathogenicity that resulted in immune responses in mice, such as M24, M25, M33, M43, and M78 [70,71,72,73,74].

## 4. Recent Studies on Salmonella-Based HIV Vaccines

### 4.1. Salmonella Strain TyCD120

A pioneer study in the development of *Salmonella*-based vector vaccines against HIV utilized an attenuated strain, dubbed TyCD120 (Table 1) [75]. The goal of this vaccine was to express the various epitopes of the gp120 viral envelope protein and induce a mucosal and systemic immune response upon oral immunization (Figure 2). The strain was transformed with an rgp120 expression cassette encoding recombinant HIV-gp120 embedded in its *aroC* locus. The expressed proteins would then be recognized by anti-gp120 antibodies to produce a response. The attenuation of TyCD120 was achieved by Δ*aroC* and Δ*aroD* deletions that are detrimental to the growth and survival of *Salmonella*, allowing for easier clearance once it has entered a host cell [76].

The gp120 monomers and multimers expressed by this attenuated *Salmonella* strain were verified using immunoblot analysis, as well as an antigen-capture ELISA that used antibodies that specifically recognized the epitopes of gp120. Although many of the epitopes were able to be recognized, antibodies that were specific to the CD4 binding domain of gp120 could not recognize the rgp120 proteins expressed by TyCD120 [75].

This study provided strong proof of concept that *Salmonella*-based delivery of vaccine components can induce strong antibody recognition but also reaffirmed the idea that the more conserved regions of the CD4 binding domain of expressed gp120 are not recognized by monoclonal antibodies.

### 4.2. aroC + Gag

Scientists in a 2009 study created a live attenuated *Salmonella*-based vaccine that targeted the Gag protein of HIV (Table 1) [77]. The Gag protein is strongly involved in the viral replication and assembly of HIV [78]. The strain used for this vaccine was a Δ*aroC* mutant that was transformed with an expression plasmid (pGEM + Gag) embedded with the HIV *gag* gene, dubbed aroC + Gag. Various assays showed that this strain was able to produce high levels of the Gag protein available for immune detection.

Mice were orally administered the aroC + Gag vaccine, showing robust immune responses [77]. These mice showed elevated levels of Gag-specific CD4+ Th1 (INF-γ and TNF-α) and Th2 (IL-4 and IL-5) cytokines. CD4+ Th1 and Th2 cells were further induced by this vaccine in the mice, as they exhibited higher levels of the Th1-related IgG2a and the Th2-related IgG1 antibodies, specific to the HIV Gag protein [77].

This study showed a strong mucosal and systemic response after oral vaccination with aroC + Gag. It showed that the attenuated *Salmonella* strain was able to adequately invade mucosal tissue and induce an HIV-specific immune response. Expression of the Gag protein by the *Salmonella* vector was also enhanced by codon-optimization, leading to increased antigen levels.

### 4.3. Ty21aBG-DNA

A 2012 study utilized an empty shell of the FDA-approved Ty21a vaccine as a delivery method for DNA vaccine components (Table 1) [79]. This “bacterial ghost” (BG) vector was generated via temperature-induced expression of the gene E from bacteriophage phi X174, leading to very high cell lysis rates, aiding in the clearance of the vectors from host cells once gene delivery is completed. The genetic material packaged inside the Ty21a vector was engineered to express gp140, a recombinant HIV envelope glycoprotein with gp120 and gp41 subunits [80]. Trimers of gp140 are of particular interest for HIV vaccine studies due to their stability and their native-like structure that mimics the antigenic properties of the immunogens found on actual HIV viral envelopes [81].

Mice were subcutaneously administered with the vaccine, dubbed Ty21a BG-DNA, twice with a two-week interval between each immunization. Results of this study showed that mice who received the vaccine showed higher levels of gp120-specific IgG1 and IgG2a antibodies, as well as increased gastrointestinal mucosal gp120-specific IgA antibodies [79]. The vaccine was also found to induce elevated production of IL-10, a Th2-associated cytokine. The Ty21a BGs may interact with the TLR4 and TLR5 pathways, potentially activating macrophages and leading to cellular activation and cytokine secretion.

### 4.4. ST-10E8

Accessibility of expressed antigens by *Salmonella* vectors has remained a point of interest among developers of these types of vaccines. A 2016 study attempted to create an attenuated *Salmonella* strain that expressed HIV antigens on the bacterium’s fimbriae so that the antigens could be more easily recognized and presented (Table 1) [82]. The attenuated strain of *Salmonella* used in this particular vaccine contained a deletion in the *aroA* gene, which is a common deletion used in attenuated strains to limit its access to certain amino acids [83]. The scientists in this study targeted highly conserved epitopes recognized by many broadly neutralizing antibodies in the HIV membrane proximal external region (MPER). To increase the immunogenicity of these epitopes, they integrated the coding sequence for the MPER epitope recognized by the 10E8 bnAb into the fimbrin gene *agfA* [82].

The researchers orally immunized mice with their ST-10E8 vaccine and observed that after 14 days, there was an increased level of serum 10E8-specific IgG (IgG1, IgG2a, and IgG2b) response, 10E8-specific mucosal secretory IgA, and strong neutralizing activity upon viral challenge with HIV. These results were further improved with a booster immunization using 10E8 peptide boosts [82]. This vaccine also induced higher levels of IL-10, IFN-γ, and TNF-α (Th1-associated cytokines), as well as higher levels of IL-4, Il-6, and IL-21 (Th2-associated cytokines). This indicates that ST-10E8 promotes B cell differentiation and antibody production.

Among the HIV candidates, the studies progressively shift from expression of relatively large viral proteins toward targeted presentation of conserved epitopes. While approaches based on the Gag and gp-140 proteins induced broad and cellular humoral responses, ST-10E8 more directly addressed HIV antigenic diversity by targeting a conserved MPER epitope recognized by broadly neutralizing antibodies.

## 5. Recent Studies on Salmonella-Based HPV Vaccines

### 5.1. Salmonella Strain SL3261

One of the first studies that created an HPV vaccine using attenuated *Salmonella* focused on the E6 and E7 proteins of HPV 16 (Table 1) [84]. E6 and E7 are oncoproteins expressed by HPV that are directly responsible for HPV-induced cancer development, as they are often continuously expressed in these tumors [85]. The attenuated strain of *Salmonella* contained a deletion in the *aroA* gene and was transformed with the pET-3xa-E6E7 expression plasmid and an invertible promoter plasmid pIP-2. These vaccine components allowed the attenuated strain of *Salmonella* to express an E6/E7 fusion protein, available for immune system recognition.

Mice were either intravenously or intradermally immunized with the vaccine and showed a clear anti-HPV antibody response 42 days after initial immunization [84]. The response was observed to grow stronger after 120 days. The *Salmonella* vectors used for this vaccine were found to have great stability, still being detected in the liver and spleen of immunized mice more than 3 weeks after immunization. The recovered vectors were also still able to produce the HPV 16 antigens. These results indicate a stable vaccine mechanism that improves immune responses with time [84].

### 5.2. Salmonella Strains PhoP^c^ L1 and PhoP^c^ L1S

A 2004 study created an attenuated vaccine strain of *Salmonella* expressing the L1 capsid protein of HPV 16 (Table 1) [86]. The HPV L1 protein spontaneously self-assembles into the icosahedral viral capsid and is a highly immunogenic structure [87]. The *Salmonella* strain PhoP^c^ contains a single point mutation in the *phoQ* gene, involved in modulating the bacterium’s virulence. This mutation results in a strain with reduced survival upon host entry, leading to quicker and more effective clearance [86]. The *asd* gene was also deleted in this strain, which compromised the integrity of the bacterium’s cell wall, further attenuating this strain [88]. The attenuated strain was transformed with a plasmid, pFS14nsd HPV16-L1S, which allowed the strain to express a recombinant L1 capsid protein.

**Table 1:** Summary of attenuated *Salmonella* vectors as vaccines against HIV, HPV, and influenza.

Pathogen	Vaccine Strain	Host	Route	Modifications	Protection/Result	Reference
HIV	TyCD120	N/A	N/A	*ΔaroC*, *ΔaroD*	N/A	[75]
	aroC + Gag	Mice	Oral	*ΔaroC*	Strong humoral response	[77]
	Ty21aBG-DNA	Mice	Subcutaneous	Empty Ty21a vector	Strong mucosal and cytokine response	[79]
	ST-10E8	Mice	Oral	*ΔaroA*	Strong mucosal and systemic humoral response	[82]
HPV	SL3261	Mice	Intravenous, Intradermal	*ΔaroA*	Adequate humoral response	[84]
	χ4550	Mice	Oral	PhoPc phenotype, *Δcya*, *Δcrp*, *Δasd*	Low humoral response	[86]
	GL04	Mice	Oral	PhoPc phenotype, ΔaroA, Δasd	Low humoral response	[86]
	pcDNA3.1-HPV16-L1	Mice	Intranasal	Empty Ty21a vector or PhoP/PhoQ vector	Humoral, cellular, and inflammatory response	[89]
Influenza	SL368	Mice	Oral	*ΔSpiR*	100%	[90]
	JOL3143	Mice	Intramuscular	*Δlon*, *ΔcpxR*, *Δasd*, *ΔsifA*	Adequate humoral response	[91]
	JOL 1913+1917	Mice	Oral, Intramuscular, Intraperitoneal	*Δlon*, *ΔcpxR*, *Δasd*, *ΔwbaP*	66% oral, 100% IM	[92]
	JOL1863	Chickens	Oral	*Δlon*, *ΔcpxR*, *Δasd*	Lower viral load	[93]
	JOL2854	Chickens	Oral	*Δlon*, *ΔcpxR*, *Δasd*, *ΔrfaL*, *ΔpagL*	80%	[94]
	rSC0130	Chickens	Oral	*ΔrelA::araC ParaBAD lacI TT*, *Δpmi*, *ΔendA::TT araC ParaBAD mazE TT*, *ΔcysG:Plac mazF*, *ΔasdA33*	75–80%	[95,96]
	χYL56	Chickens	Oral	*ΔsifA*	Lower viral load/pathology	[97]
	χYL57	Chickens	Oral	*ΔfocA*	Lower viral load/pathology	[98]

N/A, not applicable.

The researchers employed a PhoP^c^ L1 and a synthetic PhoP^c^ L1S vaccine strain for intranasal and oral vaccination in female mice [86]. The expression of the L1 and L1S proteins was measured and compared, showing higher levels of L1 expression in the PhoP^c^ L1 strain; however, much higher levels of bacteria containing L1S-encoding plasmids were recovered from mouse organs. These results indicate that the *Salmonella* strain expressing the synthetic L1S protein is more stable and lasts longer inside the host than the L1-harboring bacteria [86].

Immunization with the original PhoP^c^ L1 and synthetic PhoP^c^ L1S strains showed high IgG titers in serum, as well as HPV-specific IgG and IgA titers in vaginal washes, suggesting that the *Salmonella* vectors were immunogenic and could induce a specific immune response against HPV virus-like particles. The results of the study showed that intranasal vaccination was slightly more effective than oral vaccination and that subsequent immunizations were ineffective after 2–3 doses [86].

### 5.3. pcDNA3.1-HPV16-L1

One of the most recent studies using a *Salmonella*-based vector against HPV utilized either a Ty21a or PhoP/PhoQ vector carrying a pcDNA3.1-HPV16-L1 expression plasmid (Table 1) [89]. The plasmid, much like previous studies, expressed the highly immunogenic and self-assembling HPV 16 L1 protein. The researchers also used a PhoP/PhoQ vector to carry another plasmid, pcDNA3.1-HPV-L1-siE6, which expresses a small interfering RNA (siRNA) to silence viral expression of the oncogenic HPV proteins, E6 and E7. This would inhibit HPV’s cancer-causing effects and lead to better overall health outcomes upon HPV infection.

The results of this study showed increased levels of anti-HPV16-L1 antibodies in serum and genital secretions of mice that were intranasally immunized with both the Ty21a and PhoP/PhoQ vectors containing the pcDNA3.1-HPV16-L1 plasmid [89]. They also found that after being vaccinated three times, the immune responses in the mice showed a significant increase, including higher IL-2 and IFN-γ levels.

As for the PhoP/PhoQ strain carrying the pcDNA3.1-HPV16-L1-siE6 plasmid, mice who were vaccinated with it saw inhibited growth of SiHa cervical xenografts [89]. These results suggest that this strain of attenuated *Salmonella* was able to express the siRNA targeting the E6 and E7 proteins expressed by these HPV caused tumors. Immunohistochemical analysis of the SiHa xenografts in immunized mice showed significant down-regulation of these oncogenic proteins, further showing the growth inhibition of the tumors [89].

Although *Salmonella*-based HPV vectors demonstrate flexibility in targeting both capsid and oncogenic proteins, their range remains relatively narrow, as most studies focused on HPV16. Future multivalent constructs incorporating conserved epitopes or antigens from several high-risk HPV genotypes would therefore be necessary for these platforms to approach the wide coverage of currently licensed multivalent HPV vaccines.

## 6. Recent Studies on Salmonella-Based Influenza Vaccines

### 6.1. Salmonella Strain SL368

In 2015, a group of researchers developed an attenuated strain of *Salmonella* for an oral vaccine against the H5N1 strain of influenza (Table 1) [90]. The strain, dubbed SL368, contained a key deletion in part of the coding sequence for the SpiR protein, which is strongly linked to the PhoP/PhoQ system, which is directly involved in the regulation of SPI-2 expression [99]. The deletion resulted in more efficient gene delivery once inside the host’s cells due to the inhibition of genes responsible for the intracellular survival of *Salmonella*. This attenuated strain was then transformed with p5HA and p5NA plasmids, expressing the HA and NA genes, respectively, of H5N1 influenza [90]. The transfer of these plasmids into host cells would allow those cells to produce influenza antigens for immune system detection (Figure 1).

Mice were orally immunized with this vaccine strain, and the results were compared to the influenza vaccines that are commercially available. The SL368 vaccine showed similar levels of anti-HA serum IgG antibodies, mucosal IgA antibodies, and IFN-γ-producing T cell responses, suggesting that the vaccine induced a robust humoral and cellular response against H5N1 influenza [90].

### 6.2. JOL Strains of Salmonella

A series of *Salmonella* strains under the “JOL” name have been generated in recent years with unique mutations that enhance their function as an effective gene delivery method (Table 1) [100]. JOL1863 was a *Salmonella* strain developed in 2017 for chickens to prevent financial losses in the poultry industry due to disease [93]. The vaccine focused on the avian H7 strains of influenza (H7N3, H7N7, H7N9), and the auxotrophic strain contained a pMMP65-HA plasmid to express the influenza HA protein. The vaccine was administered intramuscularly, orally, and intranasally, with strong immune responses in each route, showing similar increases in IgG and IFN-γ. Interestingly, the oral route showed a larger increase in IgA antibodies but lower IL-10 levels, while the intramuscular group saw significantly higher levels of IL-17 [93,100].

In 2019, the attenuated strain JOL1893 was created to specifically investigate a modification focused on vector clearance from host cells, which may be an important attribute for vaccine strains so that the *Salmonella* do not persist intracellularly [101]. JOL1893 contained deletions in the *lon* and *cpxR* genes, which are virulence-associated genes [102], as well as the *asd* gene. JOL1893 contained a plasmid containing gene E from bacteriophage ϕX174, which causes the cell to lyse upon expression [103]. The expression of this gene was regulated by a λpR promoter with a thermolabile repressor cI857 that suppresses gene E at temperatures under 30 °C. Mice were vaccinated intragastrically and showed elevated levels of IgG and IgA antibodies and higher levels of IFN-γ mRNA, indicating a strong potential to stimulate T cell responses. Upon viral challenge, these mice also showed 90–100% protection against H1N1 [101].

JOL2854 was an oral vaccine strain of *Salmonella* that was created to protect chickens from the H9N2 strain of influenza (Table 1) [94]. The strain contained the same *lon*, *cpxR*, and *asd* gene deletions as the previous study but also included deletions in the *rfaL* and *pagL* genes, which play structural roles in bacterial surface lipopolysaccharides and endotoxicity of *Salmonella* [104,105]. JOL2854 was transformed with a dual-expression vector with both prokaryotic and eukaryotic promoters for an HA1:M2e sequence, which intracellularly and extracellularly expresses the influenza HA protein and the M2e protein, which is a highly conserved protein throughout avian, swine, and human strains of influenza (Table 1) [106]. The Ptrc prokaryotic promoter allowed the expression of HA1:M2e by the JOL2854 strain, resulting in MHC class II presentation upon macrophage phagocytosis of the strain. The eukaryotic promoter allowed for the host cell expression of the HA1:M2e sequence, allowing for MHC class I presentation to cytotoxic T cells, leading to a strong cellular response to the expressed antigens [94]. The vaccine showed high levels of IgA and IgY antibodies, as well as strong protection against viral challenge, as minimal gross lung pathologies and inflammatory responses were observed in chickens immunized with this vaccine.

A recent 2025 study utilized JOL1343, a *Salmonella* strain with deletions in the *lon, cpxR*, *sifA*, and *asd* genes (Table 1) [91]. The vaccine was intramuscularly administered to mice for protection against the H1N1 strain of influenza and contained a plasmid with sequences expressing highly conserved epitopes of the influenza HA and NA proteins. Vaccinated mice showed protection against lethal viral challenge, maintained body weight, and significantly reduced viral loads [91]. JOL1343 shows the potential of an epitope-based approach, as influenza inherently has variable antigens because of its rapidly evolving nature.

### 6.3. Salmonella Strain rSC0130

A 2024 study using an attenuated *Salmonella* strain known as rSC0130 focused its attention on the recruitment of immune cells toward a site of infection by utilizing an arabinose-regulated MazE/F delayed lysis system (Table 1) [95]. A plasmid with an influenza HA antigen sequence connected to a chicken C-C motif chemokine ligand 5 (chCCL5) sequence was packaged inside of rSC0130. chCCL5 is a chemokine that mainly functions to recruit many different types of immune cells in chickens [107]. Chickens orally immunized with this vaccine showed elevated Th1-associated cytokines (IFN-γ and IL-2) and Th2-associated cytokines (IL-4).

Another study using the rSC0130 *Salmonella* strain attempted to shed light on a potential avenue for a universal influenza vaccine by targeting the highly conserved M2e protein (Table 1) [96]. The mechanism of this vaccine relied on the delivery of self-assembling ferritin nanoparticles, which fused with the M2e protein, increasing the antigen’s stability and immunogenicity [108]. The vaccine was given orally to chickens, prompting a strong immune response with increased IgY antibodies specific to M2e, IL-4, and IFN-γ.

### 6.4. Salmonella Strains χYL56 and χYL57

The *sifA*-deficient χYL56 attenuated *Salmonella* strain was used in a 2025 study to express a plasmid with sequences for the N-terminus of a fused 3M2e-HA2 influenza protein and a CpG adjuvant to enhance the immune response generated by this vaccine (Table 1) [97]. The χYL56 vaccine was given orally to chickens to protect against influenza strain H9N2 and target dendritic cells. Chickens receiving this vaccine exhibited increased mRNA for IFN-γ and IL-4.

The same group conducted another study using χYL57, an attenuated *Salmonella* strain with a deletion in the *focA* gene (Table 1) [98]. The *focA* gene regulates the ability of *Salmonella* to exit macrophages and a deletion in this gene enhances this exiting ability [109]. The increase in exiting from macrophages allows *Salmonella* to potentially infect cells in other sites such as the liver or spleen. The plasmid embedded inside this *Salmonella* vector contained a sequence encoding a tetravalent influenza NA protein and a sequence for a chicken dendritic cell-targeting peptide (chDpep) [98].

In both studies, immunized chickens exhibited increased mRNAs for IFN-γ and IL-4. They also showed increased CD83, CD86, and CCR7 levels, suggesting a direct increase in dendritic cell stimulation and maturation [110,111], with the χYL57 study also showing elevated CCL5 and serum IgA production levels in immunized chickens [98]. Overall, after oral immunization, chickens showed decreased pathology and weight loss upon viral challenge with the H9N2 strains of influenza, indicating adequate protection against influenza [97,98].

### 6.5. Comparative Analysis and Broad-Spectrum Influenza Vaccine Potential

The *Salmonella*-based influenza vaccines discussed above employ substantially different strategies of selecting antigens and engineering vectors. Earlier approaches relied primarily on delivery of HA and NA antigens, whereas subsequent candidates increasingly incorporated conserved or combined influenza antigens. JOL2854 combined HA1 with the highly conserved M2e protein and utilized dual prokaryotic and eukaryotic expression to promote both MHC class I and class II antigen presentation, while JOL1343 targeted conserved HA and NA epitopes. More rSC0130 and χYL56/χYL57 platforms further incorporated conserved or multivalent antigens together with immune-modulating strategies such as ferritin nanoparticle display, CpG adjuvants, chemokine-mediated recruitment, and dendritic cell targeting.

These developments demonstrate a broader shift from strain-specific delivery toward vaccine designs intended to overcome influenza antigenic variability. Conserved epitopes are particularly attractive because they may provide immune targets that are more reliable than the highly variable regions of HA and NA. Combining multiple conserved antigens may further broaden the immune response by reducing dependence on a single viral epitope. Many of these studies tested protection against a limited number of influenza strains, and a wider range of protection is warranted, especially for studies using highly conserved antigens. A focus on cross-protection between different strains may provide vital information needed to produce a more universal vaccine that protects against influenza regardless of strain.

## 7. Advantages and Disadvantages

Most of the live attenuated *Salmonella*-based vaccine candidates discussed in this review were administered orally or intranasally. There are many advantages to these routes of administration, including easier immunization of animals, higher compliance and willingness to take the vaccines, and less waste produced. Oral and intranasal immunization does not require the use of syringes, needles, alcohol wipes, or trained clinical workers that intramuscular vaccination requires. As a result of this, there is much less biohazardous waste from many of these *Salmonella*-based vaccines.

While the human diseases outlined in this review largely already have effective treatments and vaccines, *Salmonella*-based vaccines possess the unique ability to elicit immune responses through mucosal surfaces such as the gut-associated lymphoid tissue (GALT) in the digestive tract.

### 7.1. Safety Challenges

Although *Salmonella*-based vaccines have shown great versatility and efficacy, many safety risks require careful attention.

With the numerous methods of attenuation (Table 1), over-attenuation of *Salmonella* strains must be considered. A *Salmonella* strain with too many mutations that inhibit its function may not allow the vaccine strain to carry out its intended actions, which is ultimately to enter cells to deliver genetic material for antigen expression. It may be difficult to find a careful balance between a strain that is too pathogenic or too weak. Insufficiently attenuated strains of *Salmonella* vectors may retain inflammatory potential in the intestinal mucosa [112]. Although intestinal colonization is important for the induction of mucosal immunity, excessive persistence of live attenuated *Salmonella* vectors represents a potential safety concern. Attenuated *Salmonella* strains have exhibited prolonged intestinal persistence and fecal shedding, including shedding for up to 2–3 weeks in human volunteers [113]. With the potential for increased intestinal inflammation comes an increased risk of horizontal gene transfer from vector strains of *Salmonella* to commensal bacteria of the gut. These gene transfer events may result in increased fitness, virulence, and antibiotic resistance in commensal bacteria [114].

While not all HPV-related vectors discussed in this review contain HPV oncogenes, vaccine strains that deliberately encode the viral E6 and E7 oncoproteins, such as the SL3261 strain, must prioritize retaining the functional forms of these proteins to minimize the possibility of unwanted oncogenic activity [84]. This concern may be mitigated by introducing targeted mutations that disrupt the oncogenic functions of E6 and E7 while preserving immunogenic epitopes necessary to mount an HPV-specific immune response [115].

### 7.2. Industrialization and Implementation Challenges

One of the main logistical challenges regarding live attenuated vaccines is their need for cold-chain storage and transportation. For example, the potency of the Ty21a vaccine is dependent on the temperature at which it is stored, between 2 °C and 8 °C [58]. Other processing conditions such as residual water, temperature, pressure, and excipients all factor into the potency and shelf life of this vaccine [116]. Additionally, research on room-temperature-stable bacterial vectors has shown decreased efficacy and resistance to gastrointestinal conditions [117].

While methods such as using solid forms of the Ty21a vaccine, as opposed to the liquid form, are able to better stabilize vaccine components at higher temperatures, reduce the reliance on cold-chain storage, and reduce package weight for easier distribution, the many factors at play during production, storage, and transport necessitate tight regulation and consistency of conditions [116]. Because of this, there may be high costs associated with the maintenance of ideal conditions in which to support the preservation of the vaccine. This may lead to constraints in distribution to low-resource regions, where these costs are simply too great to warrant the use of these vaccines.

## 8. Future Directions

The versatility of *Salmonella*-based vector vaccines is a powerful characteristic of this model of vaccine that can strongly aid in the fight against many diseases. While there is only one *Salmonella*-based vaccine licensed by the FDA, the promising results in animal studies have shown strong Proof of concept of their effectiveness in inducing robust immune responses. Future directions of these vaccines must include human studies to investigate their safety as well as their effectiveness in the environment of a human body. While vectors expressing bacterial antigens have been tested in humans, there remains a translational gap with virus-specific antigens. These studies must evaluate vaccine tolerance of the human gastrointestinal tract, optimal dosages and administration schedules, bacterial persistence, and strength of mucosal and systemic immune responses. Also, future research should consider the populations in which a live bacterial vector may pose a greater risk, such as those who are immunocompromised. These future studies would provide the most representative data about how effective a vaccine of this nature can be in the broader human population.

While many studies have shown that these vaccines are effective in producing the intended targeted immune responses, the exact mechanisms of gene delivery are still unclear. For example, the delivery of expression plasmids to the nucleus of a host cell to then express antigens is still not fully clarified. The need to further understand these mechanisms should not be underestimated, as this information may lead to more optimal modifications for attenuation and effectiveness. Future studies should also look to improve expression systems, whether it be bacterial expression of antigens or host-cell expression systems from delivered genetic material. With the various modifications and mechanisms at work in the studies outlined in this review, future research should continue to determine which combinations of modifications provide the best balance between safety, intracellular persistence and clearance, antigen delivery and production, and immunogenicity.

A shift towards more multivalent antigen constructs would greatly increase the viability of future vaccine studies. The use of highly conserved epitopes and emphasis on cross-protection of multiple strains of a pathogen of interest could provide much stronger protection against diverse and rapidly evolving strains such as those seen in influenza.

Longitudinal studies must also be conducted to investigate the lengths of protection that these vaccines can provide. These studies would also provide crucial information about the long-term effects of *Salmonella*-based vectors. Information from future studies will prove to be vital in swaying public opinion about live attenuated vaccines. The use of well-known pathogens such as *Salmonella* that can cause severe illness in vaccines to protect against diseases is naturally a difficult concept for the general population to grasp. This is why it is imperative to have as much information as possible to properly educate those who could most benefit from these types of vaccines.

*Salmonella*-based vector vaccines remain an evolving field with great promise. Our current review outlines several examples of *Salmonella*-based vaccine studies focusing on many prevalent viruses, specifically HIV, HPV, and influenza (Table 1). Combined with the results from previous studies, future investigations of antiviral vaccines derived from attenuated *Salmonella* will provide significant insight into our understanding of the gene delivery mechanism of *Salmonella*-based vectors and facilitate the development of novel oral vaccines against various human viruses.

## Data Availability

No new data were created or analyzed in this study. Data sharing is not applicable to this article.
